# Imaging-Based Body Fat Distribution in Polycystic Ovary Syndrome: A Systematic Review and Meta-Analysis

**DOI:** 10.3389/fendo.2021.697223

**Published:** 2021-09-09

**Authors:** Shiqin Zhu, Zeyan Li, Cuiping Hu, Fengxuan Sun, Chunling Wang, Haitao Yuan, Yan Li

**Affiliations:** ^1^School of Medicine, Cheeloo College of Medicine, Shandong University, Ji’nan, China; ^2^Center for Reproductive Medicine, Cheeloo College of Medicine, Shandong University, Ji’nan, China; ^3^National Research Center for Assisted Reproductive Technology and Reproductive Genetics, Shandong University, Ji’nan, China; ^4^Key Laboratory of Reproductive Endocrinology of Ministry of Education, Shandong University, Ji’nan, China; ^5^Department of Anesthesiology, Qilu Hospital, Cheeloo College of Medicine, Shandong University, Ji’nan, China; ^6^Department of Cardiology, Shandong Provincial Hospital Affiliated to Shandong First Medical University, Ji’nan, China; ^7^Department of Cardiology, Shandong Provincial Hospital, Cheeloo College of Medicine, Shandong University, Ji’nan, China; ^8^Suzhou Research Institute, Shandong University, Suzhou, China

**Keywords:** body fat distribution, central obesity, imaging method, polycystic ovary syndrome, systematic review and meta-analysis

## Abstract

**Background:**

Women with polycystic ovary syndrome (PCOS) are generally considered to be central obese and at higher risks of metabolic disturbances. Imaging methods are the golden standards for detecting body fat distribution. However, evidence based on magnetic resonance imaging (MRI) and computed tomography (CT) is conflicting. This study systematically reviewed the imaging-based body fat distribution in PCOS patients and quantitatively evaluated the difference in body fat distribution between PCOS and BMI-matched controls.

**Methods:**

PUBMED, EMBASE, and Web of Science were searched up to December 2019, and studies quantitatively compared body fat distribution by MRI, CT, ultrasound, or X-ray absorptiometry (DXA) between women with PCOS and their BMI-matched controls were included. Two researchers independently reviewed the articles, extract data and evaluated the study quality based on Newcastle-Ottawa Scale (NOS).

**Results:**

47 studies were included in systematic review and 39 were eligible for meta-analysis. Compared to BMI-matched controls, higher accumulations of visceral fat (SMD 0.41; 95%CI: 0.23-0.59), abdominal subcutaneous fat (SMD 0.31; 95%CI: 0.20-0.41), total body fat (SMD 0.19; 95% CI: 0.06-0.32), trunk fat (SMD 0.47; 95% CI: 0.17-0.77), and android fat (SMD 0. 36; 95% CI: 0.06-0.66) were identified in PCOS group. However, no significant difference was identified in all the above outcomes in subgroups only including studies using golden standards MRI or CT to evaluate body fat distribution (SMD 0.19; 95%CI: -0.04-0.41 for visceral fat; SMD 0.15; 95%CI: -0.01-0.31 for abdominal subcutaneous fat). Moreover, meta-regression and subgroup analyses showed that young and non-obese patients were more likely to accumulate android fat.

**Conclusions:**

PCOS women seem to have abdominal fat accumulation when compared with BMI-matched controls. However, MRI- and CT- assessed fat distribution was similar between PCOS and controls, suggesting central obesity may be independent of PCOS. These findings will help us reappraise the relationship between PCOS and abnormal fat deposition and develop specialized lifestyle interventions for PCOS patients.

**Systematic Review Registration:**

PROSPERO, identifier CRD42018102983.

## Introduction

Polycystic ovary syndrome (PCOS) is an endocrine disease associated with obesity and multiple metabolic complications, including insulin resistance, diabetes, and cardiovascular diseases (1, 2). According to previous studies, metabolic disturbances in PCOS are partially obesity-related conditions (3). It has been widely acknowledged that obesity aggravates insulin resistance and adverse metabolic outcomes in patients with PCOS (4). However, there are approximately 40–50% of PCOS patients with BMI in the normal range (5). These lean PCOS patients also have increased risks of metabolic dysfunctions and merely losing weight is not a suitable intervention for this population (6). Thus, it is important to investigate whether the body composition and body fat distribution are altered in PCOS patients since different body compositions (that is different percentages of fat, muscle and bone, and body fat mass) may be completely different under the same BMI.

According to the World Health Organization, body fat distribution is another factor that determines the metabolic risks associated with obesity (7). Visceral fat and abdominal subcutaneous fat, which are known as android fat, are recognized to be related to higher risks of metabolic abnormalities such as hypertension and type 2 diabetes, while gluteal or thigh fat, known as gynoid fat, is regarded as a protective fat correlated with low risks of metabolic diseases (8). Different methods can be used to measure fat distribution. Waist circumference (WC), as a conventional clinical measurement of abdominal obesity, has been widely used to estimate central obesity in PCOS patients. A previous meta-analysis showed that women with PCOS had a higher prevalence of central obesity according to WC (9). The golden standard for the measurement of body fat distribution are imaging methods such as magnetic resonance imaging (MRI) and computed tomography (CT). However, studies using these methods to assess fat distribution in women with PCOS showed controversial results. A study using MRI to analyze body composition of women with or without PCOS argued that lean women with PCOS had less visceral fat (10), whereas two other studies including MRI assessment reported no visceral fat accumulation in PCOS women with obesity or insulin resistance (11, 12).

Therefore, it is essential to quantitatively study body fat deposition of PCOS through imaging methods to help us get in-depth knowledge of the fat distribution of women with PCOS. This study systematically reviewed the imaging-based body fat distribution in PCOS patients and quantitatively evaluated the difference between PCOS and BMI-matched controls from 8 aspects: visceral fat, abdominal subcutaneous fat, total body fat, trunk fat, android fat, and gynoid fat. Our findings provide new insights into the fat distribution patterns in PCOS patients, which is of great significance for understanding the etiology of PCOS and guiding lifestyle interventions in clinical practice.

## Material and Methods

### Search Strategy

Systematic database searches were performed in PUBMED, EMBASE, and Web of Science updated in Dec 2019. The declarations of Preferred Reporting Item for Systematic Reviews and Meta-analyses (PRISMA) were followed. The protocol of this systematic review and meta-analysis was previously registered on PROSPERO (CRD42018102983). We developed the “full text” search strategy based on the combination of the following keywords (subject item plus free items): (polycystic ovary syndrome OR PCOS) AND (body fat distribution OR visceral adipose tissue OR subcutaneous adipose tissue OR central obesity OR android distribution OR gynoid distribution) AND (magnetic resonance OR ultrasound OR computerized tomography OR X-ray). Detailed search strategies were listed in **Table S1**. Full-text review was implemented after the screening of title and abstract. References of included articles were hand-reviewed to identify the eligible articles.

### Inclusion and Exclusion Criteria

The populations being studied in this review were women diagnosed with PCOS and the populations of comparator were BMI-matched control women without PCOS. This review was based on observational studies, therefore interventions were not applicable. The main outcomes in this study were imaging-based body fat distribution including visceral fat, abdominal subcutaneous fat, total body fat, trunk fat, android fat, and gynoid fat in quantity. Moreover, we also included total body fat, trunk fat, android fat, and gynoid fat in percentage as secondary outcomes. Studies that satisfied the following criteria were included in the present meta-analysis: (1) studies that investigated the distribution of body fat including visceral fat, abdominal subcutaneous fat, total body fat (both in quantity and in percentage), android fat (both in quantity and in percentage), gynoid fat (both in quantity and in percentage), and trunk fat (both in quantity and in percentage) between women with PCOS and controls; (2) body fat distribution was measured by standard imaging methods including MRI, CT, ultrasound, and X-ray absorptiometry (DXA); (3) When duplication of same subject population occurred, the most recent study or study with the largest sample was included. Exclusion criteria were: (1) studies that employed testing technologies other than the standard imaging methods such as bioelectrical impedance; (2) studies without BMI adjustment; (3) studies that lack sufficient data to perform quantitative or qualitative analysis. Articles in languages other than English were excluded.

### Data Extraction

Two researchers (Zhu SQ and Hu CP) reviewed retrieved studies independently. Baseline characteristics relating to study and its participant (country, ethnicity, design, age, BMI, definition of PCOS, subject number, adjusted confounders, imaging methods, treatments, blind to outcomes, measure region, outcomes) were extracted. The definition of PCOS was defined by Rotterdam criteria, National Institutes of Health (NIH) criteria, the Androgen Excess and PCOS (AE-PCOS) Society criteria, or based on the original articles (13–15). Definitions of obese and non-obese were arbitrarily decided based on original articles due to the heterogeneity of cutoff values. Disagreements were resolved after consensus (Li Y). Regarding the studies with insufficient data and some conference abstracts, the corresponding authors were contacted.

### Quality Assessment

Newcastle-Ottawa Scale (NOS) was employed to assess the qualities of the included studies (16). Two researchers (Zhu SQ and Li ZY) independently evaluated the study quality in a blinded manner. Controversies were settled by consultation among co-authors. NOS focuses on three aspects of quality assessment: (1) selection of representative cases and controls; (2) comparability of baseline features; (3) exposure assessment or outcome evaluation. NOS score with minimum 0 and maximum 9, which higher score indicates higher quality.

### Statistical Analysis

We evaluated the variation of fat distribution between women with PCOS and controls. The between-study standardized mean difference (SMD) and 95% confidence intervals (CIs) were deployed. SMD>0 represents higher fat distribution in PCOS as compared to that of controls, while SMD<0 indicated the opposite. Between-study heterogeneity was assessed by I2-statistics and Q-test. Random-effects model was used when significant heterogeneity was observed. Subgroup analyses were performed to explore the source of heterogeneity. The impact of a study on the overall effect was assessed through sensitivity analysis. Besides, meta-regression was performed on outcomes that have more than 10 included studies. Publication bias was examined using Egger’s regression test. Two-tailed P < 0.05 was considered as significant. Trim and Filled Analysis was used to further evaluate the variation when significant bias was tested in Egger’s regression test. Statistical analyses were performed using STATA 12.0 (Stata Corporation, College Station, USA).

## Results

### Search Results

**Figure 1:**
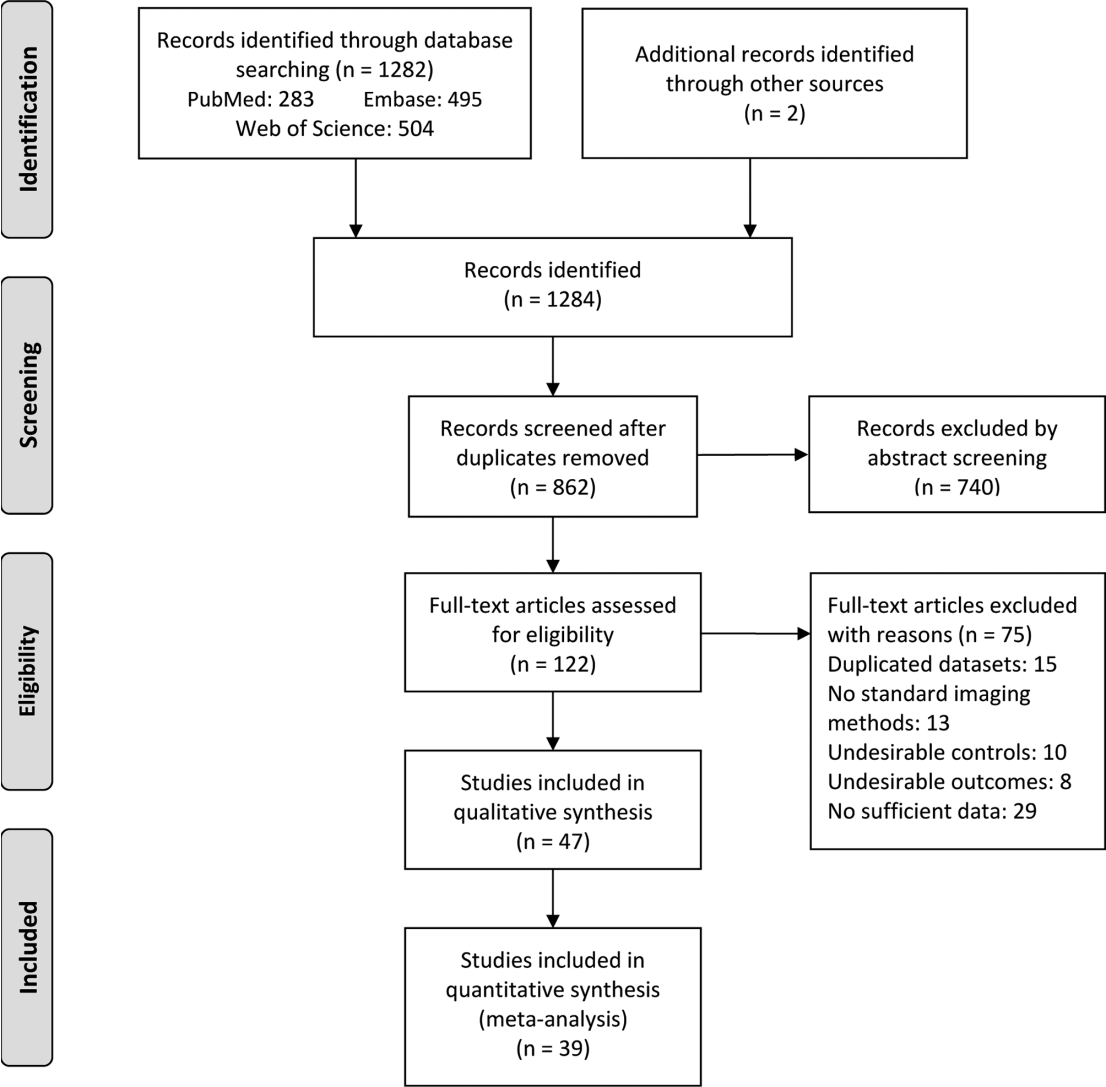
PRISMA flow diagram for study selection. From: Moher D, Liberati A, Tetzlaff J, Altman DG, The PRISMA Group (2009). Preferred Reporting Items for Systematic Reviews and Meta-Analyses: The PRISMA Statement. PLoS Med 6(7): e1000097. doi:10.1371/journal.pmed1000097.

A total of 1284 articles were yielded through electronic search strategy and hand search. After excluding duplicates and screening abstracts based on selection criteria, 122 full-text articles were further assessed for eligibility. After excluding 15 for duplicated datasets, 13 for not using standard imaging methods, 29 for insufficient data, 18 for undesirable controls or outcomes, 47 articles finally remained for qualitative synthesis and 39 were found eligible for meta-analysis (**Figure 1**).

### Study Characteristics

This study included overall 4226 individuals, 2203 with PCOS and 2023 controls (**Table 1**). BMI was similar between PCOS group and control group in every eligible study. Fat distribution was measured in all study subjects using imaging methods. Among these studies, 10 used MRI (10–12, 17, 18, 23–27), 7 used CT (19–22, 28–30), 8 used ultrasound (31–38), and 30 used DXA (23–30, 39–60) as their measurement indicator. Details of detected areas in each study were displayed in **Table 1**. The ethnicity of eligible studies varied from Caucasian, Asian, and Mediterranean. Diagnostic criteria of PCOS were adopted NIH, Rotterdam, or AE-PCOS criteria in most of the included studies, and 5 studies describe PCOS definition in their original articles (**Table S2**).

**Table 1:** Study characteristics of included studies.

Author	Year	Country	No. of Participants		Age (year) Mean#/Median*		BMI (kg/m2) Mean/Median		Imaging measure	Outcomes (Region)	Results^b^ (PCOS *VS* Control)	NOS	Included in meta analysis
			PCOS	Control	PCOS	Control	PCOS	Control					
Barber et al	(11)	UK	22	22	# 30.3 ± 5.6	# 39.5 ± 6.1	*28.2 (22.4, 35.5)	*28.3 (22.8, 35.1)	MRI	Visceral fat (Mid-L4) Abdominal sc fat (Mid-L4) Gynoid fat (mid-thigh)	No significant differences	8	YES
Diaz et al	(17)	Spain	35	25	# 15.8 ± 0.2	# 15.6 ± 0.2	# 23.6 ± 0.5	# 22.2 ± 0.5	MRI	Visceral fat (L4–L5) Abdominal sc fat (L4–L5)	Visceral fat: increased (P<0.01) Abdominal sc fat: increased (P<0.01)	8	YES
Dolfing et al	(10)	Netherlands	10	10	# 28.2 ± 2.6	# 33.7 ± 2.3	#21.6±1.1	# 21.8± 2.1	MRI	Visceral fat (Mid-L4) Abdominal sc fat (Mid-L4)	No significant differences	6	YES
Jones et al	(18)	UK	29	22	*28 (26–31)	*29 (28–31)	*33 (31–36)	*30 (28–33)	MRI	Visceral fat (abdominal region) Abdominal sc fat (abdominal region) Total body fat	Visceral fat: increased (P=0.05)	8	YES
Mannerås-Holm et al	(12)	Sweden	31	31	# 28.5 ± 3.6	# 27.8 ± 3.5	# 24.8±4.8	# 24.7± 4.9	MRI	Visceral fat (L4–L5) Abdominal sc fat (L4–L5) Android fat (abdominal region)	No significant differences	9	YES
Ezeh et al	(19)	US	16	16	# 29.2±0.9	# 33.9±1.3	# 32.3±1.4	# 29.5±1.21	CT	Visceral fat (L4–L5) Abdominal sc fat (L4–L5)	No significant differences	8	YES
Jin et al	(20)	Korean	90	97	#26.3 ± 6.3	#31.4 ± 6.5	#23.3 ± 4.9	#22.2 ± 3.65	CT	Visceral fat (L4–L5) Abdominal sc fat (L4–L5) Android fat (abdominal region) Gynoid fat (mid-thigh)	No significant differences	6	YES
Pasquali et al	(21)	Italy	Group 1: 12 Group 2: 8	Group 1: 8 Group 2:12	#Group 1: 30.8±7.4 Group 2: 32.3±5.0	#Group 1: 31.6±10.3 Group 2: 36.3±9.5	# Group 1: 39.8±7.9 Group 2: 39.6±6.9	# Group 1: 37.4±3.0 Group 2: 40.1±6.2	CT	Visceral fat (L4–L5) Abdominal sc fat (L4–L5) Total body fat	No significant differences	8	YES
Penaforte et al	(22)	Brasil	30	15	# 30.5 ± 5.0	# 32.3 ± 5.6	# 36.3 ± 4.1	# 35.2 ± 4.7	CT	Visceral fat (L4–L5) Android fat (L4–L5) Trunk fat (L3)	No significant differences	7	YES
Boumosleh et al	(23)	US	Nonobese: 37 Obese: 108	Nonobese: 107 Obese: 237	*Nonobese: 40 (38, 41) Obese: 41 (39, 42)	*Nonobese: 41 (39, 42) Obese: 42 (41, 43)	*Nonobese: 23.9 (23.0, 25.5) Obese: 33.9 (32.5, 36.9)	*Nonobese: 23.9 (22.8, 24.9) Obese: 32.1 (30.9, 33.3)	DXA,MRI	Visceral fat (L2–L3) Abdominal sc fat (L2-L3) Total body fat^a^ Trunk fat^a^ (below the chin to the pubic symphysis)	Trunk fat^a^: increased in obese group (P<0.05) Abdominal sc fat: increased in obese group (P<0.05)	5	NO
Dumesic et al	(24)	USA	6	14	# 25.3±1.8	# 26.9±1.4	# 21.8±0.8	# 21.7±0.5	DXA, MRI	Visceral fat (T12-L5) Abdominal sc fat (T12-L5) Total body fat^a^ Android fat ^a^ (L1 to the pelvis) Gynoid fat^a^ (head of the femur to midthigh)	Android fat^a^: increased (P=0.02) Visceral fat: increased (P=0.03)	9	YES
Echiburú et al	(25)	Chile	12	12	*37.5 (31.0 – 42.0)	*42.5 (38.7 – 43.7)	*28.5 (26.1 – 32.6)	*26.3 (23.7– 29.1)	DXA,MRI	Visceral fat (L4–L5) Abdominal sc fat (L4 –L5) Total body fat^a^ Trunk fat^a^ (NA)	Trunk fat (kg): increased (P=0.043)	6	NO
Huang et al	(26)	USA	14	14	# 26±5	# 27±6	# 27.2±4.1	# 25.5±3.9	DXA, MRI	Visceral fat (L3) Abdominal sc fat (L3) Trunk fat^a^ (NA)	Trunk fat (%):increased (P=0.04) Visceral fat: increased (P=0.009) Subcutaneous: increased (P=0.005)	7	NO
Ibáñez et al	(27)	Spain	9	29	15	15	NA	NA	DXA, MRI	Visceral fat (abdominal region) Abdominal sc fat (abdominal region) Total body fat Android fat^a^ (NA)	Android fat (%): increased (P <0.001)	9	YES
Hutchison et al	(28)	Australia	20	14	# 29.5±1.4	# 35.0±1.1	# 37.4±1.5	# 35.7±1.3	DXA,CT	Visceral fat (L4-L5) Abdominal sc fat (L4-L5) Android fat (abdominal region) Total body fat	Visceral fat: increased (P=0.04)	7	YES
Kim et al	(29)	US	21	21	# 13.6 ± 2.2	# 13.3 ± 2.2	# 32.7 ± 4.5	# 33.3 ± 5.4	DXA,CT	Visceral fat (L4-L5) Total body fat^a^	Visceral fat: increased (P=0.011)	7	YES
Morrison et al	(30)	USA	30	38	#31.6±5.7	#34.8±8.7	# 31.8 ± 5.7	# 31.5 ± 4.9	DXA,CT	Visceral fat (L4-L5) Abdominal sc fat (L4-L5) Gynoid fat (mid-thigh) Total body fat	No significant differences	7	YES
Borruel et al	(31)	Spain	55	25	# 26±6	# 30±5	# 30.5±8.8	# 27.4±7.5	Ultrasound	Abdominal sc fat (umbilicus)	No significant differences	7	YES
Cascella et al	(32)	Italy	200	100	#24.6±3.2	#24.0±2.8	#28.5±2.8	#28.8±2.7	Ultrasound	Visceral fat (1 cm above the umbilicus)	Visceral fat: increased (P=0.001)	6	YES
Jena et al	(33)	India	58	40	# 21.86±5.22	# 22.72±5.11	# 28.14±5.94	# 27.0±7.08	Ultrasound	Visceral fat (linea alba to lumbar vertebra) Abdominal sc fat (cutaneous boundary to linea alba)	Visceral fat: increased (P=0.003) Abdominal sc fat: increased (P=0.014)	8	YES
Karabulut et al	(34)	Turkey	46	43	#25.9 ± 5.5	#25.2 ± 5.0	#27.5 ± 3.9	#26.6 ± 3.8	Ultrasound	Visceral fat (1 cm above the umbilicus) Abdominal sc fat (midline of abdomen) Gynoid fat (mid-thigh)	Abdominal sc fat: increased (P=0.01) Visceral fat: increased (P<0.01)	9	YES
Moran et al	(35)	Mexico	Obese:69 Nonobese:67	Obese:9 Nonobese:33	#Obese:27.6±3.4 Nonobese:27.6±3.7	#Obese:29.4±4.4 Nonobese:28.5±4.0	#Obese:34.6±3.4 Nonobese:27.0±2.3	#Obese:35.3±4.8 Nonobese:24.9±2.2	Ultrasound	Visceral fat (posterior aponeurosis of rectus abdominis to the anterior wall of the aorta) Abdominal sc fat (5 cm above the navel)	Abdominal sc fat: increased in non-obese group (P<0.05)	8	YES
Sahin et al	(36)	Turkey	Obese: 33 Non-obese: 35	Obese: 16 Non-obese: 24	# Obese: 22.1 ± 4.3 Non-obese: 20.4 ± 3.2	# Obese: 22.6 ± 4.3 Non-obese: 22.04 ± 4.8	# Obese: 37.2 ± 6.7 Non-obese: 23.7 ± 2.7	# Obese: 36.4 ± 4.5 Non-obese: 22.5 ± 2.9	Ultrasound	Abdominal sc fat (lateral abdomen)	No significant differences	6	YES
Tripathy et al	(37)	India	124	177	# 27.22±4.76	# 26.81±4.75	# 25.98 ± 3.14	# 25.86±3.31	Ultrasound	Visceral fat (umbilicus) Abdominal sc fat (umbilicus)	Visceral fat: increased (P=0.01)	8	YES
Yildirim et al	(38)	Turkey	30	30	# 27.7±6.0	# 29.5±6.0	# 19.7±2.4	# 20.3±2.7	Ultrasound	Visceral fat (internal face of the abdominal muscle to the anterior wall of the aorta) Abdominal sc fat (upper median abdomen)	Visceral fat: increased (P<0.01)	7	YES
Braga et al	(39)	Brazil	30	28	# 27.8±6.5	# 28.8±5.8	26.8 (22.3±33.4)	26.3 (23.4±32.8)	DXA	Android fat (%) (NA) Gynoid fat (%) (NA) Trunk fat (%) (NA)	Android fat (%): increased (P=0.04) Trunk fat (%): increased (P=0.03)	7	YES
Carmina et al	(40)	Italy	Obese:35 Overweight:35: Normoweight: 40	Obese:36 Overweight:36: Normoweight: 40	#25.1±4.9	#24.9±3	# Obese:34.4±3.8 Overweight:27.8±1.1: Normoweight: 27.8±1.1	# Obese:34.5±3.8 Overweight:27.8±1.8 Normoweight: 22.4±1.5	DXA	Trunk fat^a^ (NA) Android fat^a^ (between the lateral iliac crests and the lowest rib margins) Total body fat	Trunk fat (%): increased in normoweight group (P<0.01) Android fat^a^: increased in normoweight and overweight group (P<0.01)	7	YES
Cree-Green et al	(41)	US	18	20	# 15.9 ±1.83	# 15.0 ±2.13	# 22.7±2.3	# 21.3± 2.9	DXA	Total body fat (%)	No significant differences	6	NO
Cree-Green et al	(42)	US	41	30	*15.0 (13.00,16.00)	*14.5 (13.00,17.00)	#35.2±0.61	# 33.2±1.79	DXA	Total body fat (%)	No significant differences	6	NO
Cunha et al	(43)	Brazil	39	34	# 25.17±3.86	# 25.67±4.42	*24.43 (20.90-33.84)	*23.95 (21.62-31.0)	DXA	Total body fat (%) Trunk fat (%) (below the chin to colli femori) Android fat (%) (superior iliac spines to 20% above)	No significant differences	8	YES
Faloia et al	(44)	Italy	Lean: 23, overweight/obese:27	lean: 12, overweight/obesel:8	#Lean:23±5, overweight/obese:21±5	#lean: 26±3, overweight/obese:24.7±4.8	#Lean:22±2, overweight/obese:32±5	#lean: 20±1.3, overweight/obese:37±5.9	DXA	Android fat (%) (L2-L4) Total body fat (%)	No significant differences	6	YES
Glintborg et al	(45)	Denmark	167	110	*30(25–33)	*30(25–36)	*28.6(23.8–32.2)	*26.7(22.9–30.4)	DXA	Trunk fat (below the chin to colli femori) Total body fat^a^	Trunk fat: increased (P=0.07)	8	NO
Godoy-Matos et al	(46)	Brazil	24	13	# 28.3±8.4	# 28.1± 8.7	# 78.8 ±21.2	# 76.2 ±20.1	DXA	Trunk fat (%) (NA)	No significant differences	8	YES
González et al	(47)	USA	Normal-Weight , Normal AA:7 NormalWeight, Excess AA:8 Obese :8	Normal-Weight , Normal AA:8 Normal-Weight , Excess AA:8 Obese :8	# Normal-Weight , Normal AA: 24 ± 2 Normal-Weight , Excess AA: 29 ± 1 Obese : 27 ± 2	# Normal-Weight , Normal AA: 30 ± 3 Normal-Weight , Excess AA: 29 ± 1 Obese : 30 ± 3	# Normal-Weight , Normal AA: 22.2 ± 0.9 Normal-Weight , Excess AA: 24.3 ± 0.4 Obese: 37.1 ± 0.9	# Normal-Weight , Normal AA: 22.2 ± 0.5 Normal-Weight , Excess AA: 22.8 ± 0.5 Obese: 35.8±0.9	DXA	Trunk fat^a^ (dome of the diaphragm to the top of the great trochanter) Total body fat	Trunk fat (kg): increased in obese group (P<0.04) Trunk fat (%): increased in normal-weight, excess AA group (P<0.008)	7	YES
Good et al	(48)	US	12	10	# 28.5± 7.0	# 28.9 ± 8.3	# 22.4 ± 2.3	# 22.0 ± 2.2	DXA	Trunk fat (%) (NA) Total body fat (%)	No significant differences	7	YES
Jedrzejuk et al	(49)	Poland	62	43	# 24.2±4.8	# 26.7±6.5	# 22.0±1.4	# 22.5±2.0	DXA	Total body fat (%) Android fat (%) (L2-L4) Gynoid fat (%) (NA)	Total body fat (%) : increased (P=0.014) Android fat (%): increased (P=0.013)	7	YES
Kirchengast et al	(50)	Austria	16	19	NA	#23.7	# 21.51±1.42	# 20.61±2.37	DXA	Trunk fat (below the chin to colli femuri) Total body fat	Trunk fat: increased (P<0.01) Total body fat: increased (P<0.01)	6	YES
Kogure et al	(51)	Brazil	Normoweight: 13; Overweight: 10; Obese: 17;	Normoweight: 16; Overweight: 13; Obese: 11;	*26.8 (18.6–37.3)	*28.2 (20.4–30.7)	*28.9 (19.5–39.6)	*26.9 (18.9–40.0)	DXA	Total body fat (%) Android fat (%) (NA) Gynoid fat (%) (NA)	Android fat (%): increased in normoweight group (P<0.05) Gynoid fat (%): increased in normoweight group (P<0.05)	7	NO
Macruz et al	(52)	Brazil	28	16	#24.7±7.3	#23.3±7.1	#21.7±2.1	#21.1s±2.3	DXA	Trunk fat (%) (NA) Total body fat (%)	Trunk fat (%): increased (P=0.001)	7	YES
Mierzwicka et al	(53)	Poland	73	61	# 24.3 ± 4.8	# 29.2 ± 6.4	# 27.5 ± 6.4	# 25.3 ± 4.7	DXA	Android fat (%) (NA) Gynoid fat (%) (NA) Total body fat (%)	Android fat (%): increased (P<0.01)	6	YES
Pepene et al	(54)	Romania	50	17	#26.660 ± 1.018	#29.882 ± 1.945	# 33.509 ± 0.777	# 31.838 ± 0.985	DXA	Total body fat	No significant differences	6	YES
Satyaraddi et al	(55)	India	42	42	# 25.2±3.9	# 25.3±3.8	# 30.9±4.9	# 29.6±4.4	DXA	Visceral fat (NA) Total body fat^a^	Visceral fat: increased (P=0.0001) Total body fat (%): increased (P=0.0001)	8	YES
Schmidt et al	(56)	Sweden	20	66	*68.0 (61.0-78.0)	*68.5 (61.0-80.0)	*27.7 (20.9-37.7)	*25.7 (16.9-40.1)	DXA	Total body fat	No significant differences	6	NO
Shroff et al	(57)	USA	24	24	# 32 ±6.5	# 36 ± 7.2	# 36 ±5.4	# 35 ± 3.3	DXA	Trunk fat (%) (standardized region) Total body fat	No significant differences	8	YES
Thomann et al	(58)	Switzerland	20	19	# 28.0 ± 5.8	# 30.8± 4.0	# 26.3 ± 5.7	# 25.0± 4.5	DXA	Trunk fat (%) (NA) Total body fat (%)	Trunk fat (%): increased (P=0.002)	6	YES
Toscani et al	(59)	Brazil.	24	13	#23 ± 1.4	#27 ± 2.5	#34 ± 1	#30.2 ± 1	DXA	Trunk fat (total body fat mass minus the arms and legs fat mass) Total body fat (%)	Trunk fat: increased (P=0.038)	7	YES
Yucel et al	(60)	Turkey	33	21	# 27.6±3.9	# 29.1±3.1	# 27.41±5.76	# 26.03±4.81	DXA	Trunk fat (below the chin to the colli femuri) Total body fat	Trunk fat: increased (P< 0.043)	8	YES

Total body fat, trunk fat, android fat and gynoid fat could be expressed in quantity or in percentage. Outcomes marked with (%) refer to outcomes expressed in percentage, and outcome without (%) mark refer to outcomes expressed in quantity.

No., Number; DXA, Dual X-ray absorpsiometry; Abdominal sc fat, Abdominal subcutaneus fat; NA, Not available.

^a^both in quantity and percentage; ^b^Only displayed the outcomes with significant results in PCOS compared to BMI-matched control. ^#^represents means and *represents medians.

### Methodological Quality

Assessments of study quality were displayed in **Table S3**. All studies were ranked into medium or high quality, except for one study that was graded low quality (excluded in the meta-analysis). Among all 47 studies, 44 adjusted other confounders such as age, weight, or ethnicity. 35 studies clarified the age stage of participants, of which 25 studies investigated fat distribution in adults. 25 studies stratified participants into specified BMI categories. Overall 43 studies reported no medications or treatment interferences and 11 declared as a blinded study (**Table S2**). Studies whose data fit the normal distribution and expressed as mean were included in the meta-analysis, whereas studies with data displayed as median were only included in the systematic review.

### Visceral Fat and Abdominal Subcutaneous Fat

**Figure 2:**
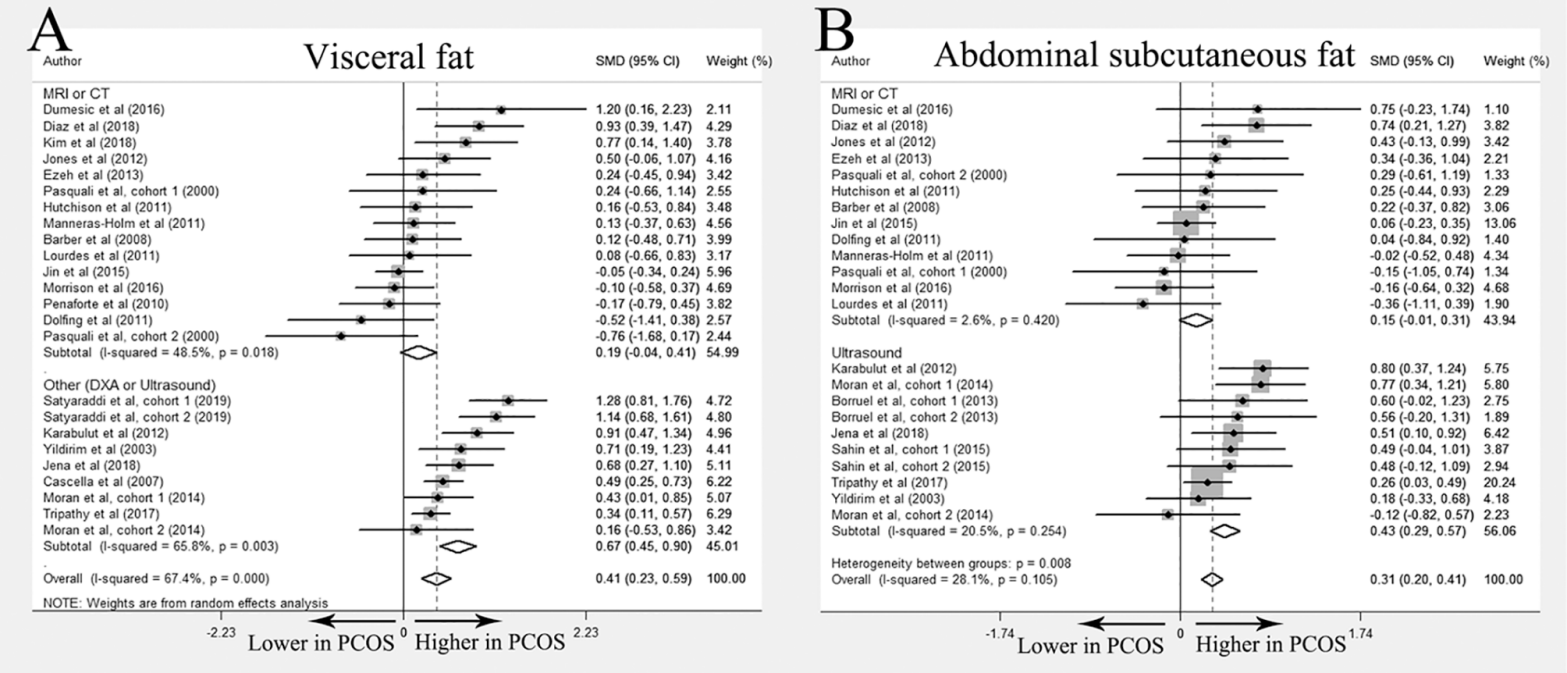
Meta-analysis on visceral fat and abdominal subcutaneous fat: women with PCOS *versus* BMI-matched healthy controls. Forest plot displayed odds of visceral fat **(A)** and abdominal subcutaneous fat **(B)** in subgroups.

Overall 24 studies (10–12, 17–30, 32–35, 37, 38, 55) compared the difference of visceral fat between women with PCOS and healthy controls and 21 studies (10–12, 17–22, 24, 27–30, 32–35, 37, 38, 55) were included in meta-analysis. Most studies found no differences in fat distribution between PCOS patients and controls. Huang et al. demonstrated increased visceral fat in PCOS (26), whereas Boumosleh et al. and Echiburú et al. reported similar visceral fat distribution between two groups (23, 25). In the meta-analysis, increased visceral fat accumulation was identified in women with PCOS (SMD 0.41; 95%CI: 0.23-0.59). However, this difference disappeared when imaging methods were restricted to MRI or CT (SMD 0.19; 95%CI: -0.04-0.41) (**Figure 2A**).

Twenty-two studies investigated abdominal subcutaneous fat distribution in PCOS and control groups (10–12, 17–21, 23–28, 30, 31, 33–38). Among them, 5 studies reported elevated abdominal subcutaneous fat accumulation in the PCOS group (17, 23, 33–35), and no significant differences were reported in the remaining studies. Meta-analysis of abdominal subcutaneous fat included 19 studies (10–12, 17–21, 24, 27, 28, 30, 31, 33–38). The results showed that women with PCOS had more abdominal subcutaneous fat than their BMI-matched healthy controls (SMD 0.31; 95%CI: 0.20-0.41). However, similar to the results of visceral fat, no significant differences were found in the subgroup including only studies using MRI or CT (SMD 0.15; 95%CI: -0.01-0.31) (**Figure 2B**).

### Total Body Fat, Trunk Fat, Android Fat and Gynoid Fat

A total of 37 (11, 12, 18, 20–30, 34, 39–60) studies compared the distribution of total body fat, trunk fat, android fat, and gynoid fat between PCOS and BMI-matched control groups, and each outcome was expressed as quantity and percentage. Most of these studies used DXA as the imaging method, especially when describing the percentage of fat distribution. Detailed information about each study was displayed in **Table 1**. Overall 29 studies (11, 12, 18, 20–22, 24, 27–30, 34, 39, 40, 43, 44, 46–50, 52–55, 57–60) were further included in the meta-analysis. Of them, 23 investigated total body fat distribution (N=14 for quantity; N=11 for percentage); 13 studies trunk fat distribution (N=6 for quantity; N=9 for percentage); 12 studies android fat distribution (N=7 for quantity; N=8 for percentage); and 8 studied gynoid fat distribution (N=5 for quantity; N=4 for percentage).

**Figure 3:**
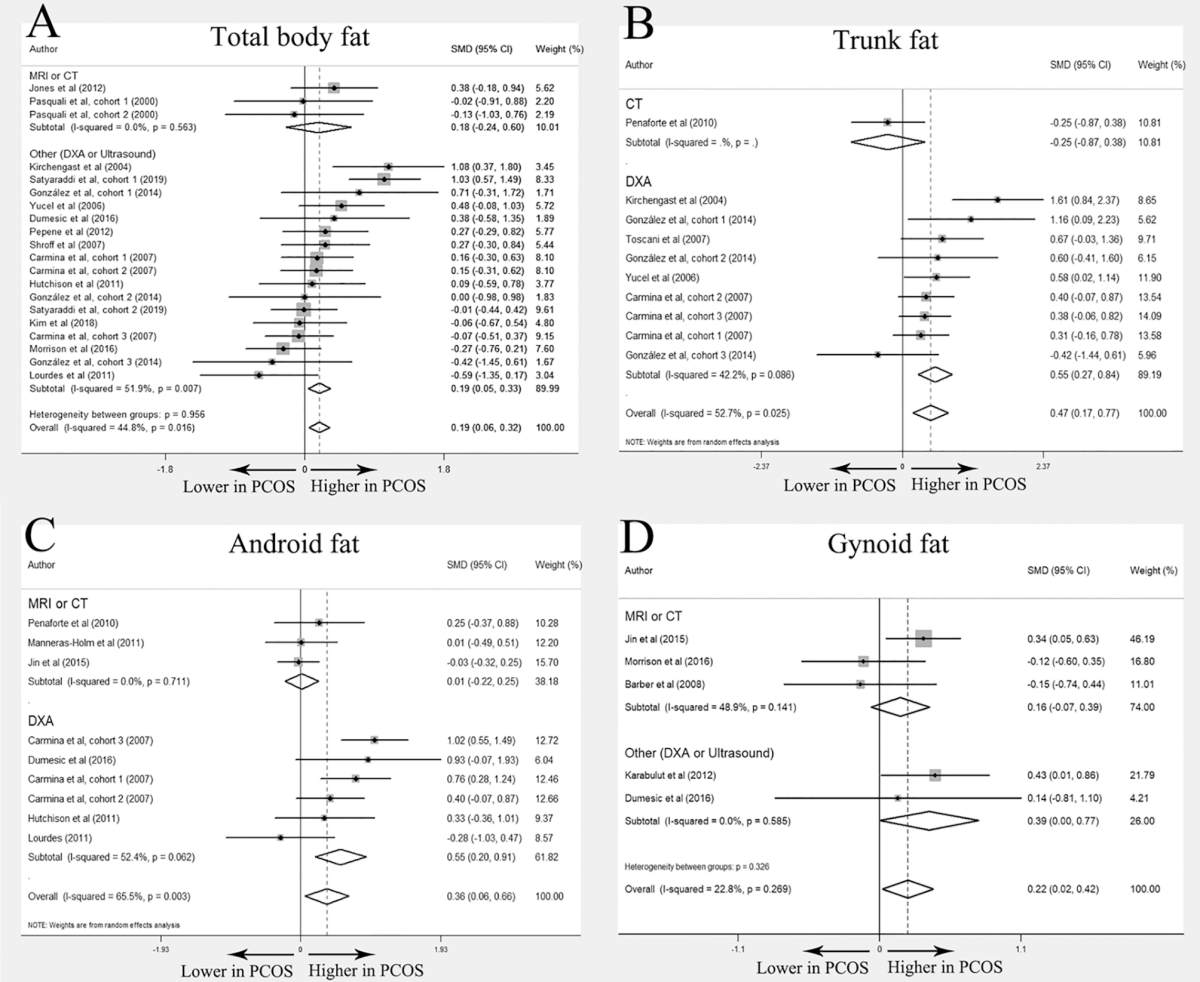
Meta-analysis on total body fat, trunk fat, android fat and gynoid fat (in quantity): women with PCOS *versus* BMI-matched healthy controls. Forest plot displayed odds of total body fat **(A)**, trunk fat **(B)**, android fat **(C)** and gynoid fat **(D)** in subgroups.

In the meta-analysis, absolute value of total body fat was elevated in PCOS women but no significant difference was found in the percentage of total body fat between women with PCOS and BMI-matched controls (SMD 0. 19; 95% CI: 0.06-0.32 for quantity; SMD 0.27; 95% CI: -0.14-0.69 for percentage). Increased accumulation of trunk fat (SMD 0.47; 95% CI: 0.17-0.77 for quantity; SMD 0.67; 95% CI: 0.40-0.94 for percentage) and android fat (SMD 0. 36; 95% CI: 0.06-0.66 for quantity; SMD 0.53; 95% CI: 0.12-0.94 for percentage) was identified in women with PCOS. PCOS women also had higher absolute value of gynoid fat (SMD 0. 22, 95% CI: 0. 02-0. 42 for quantity), whereas the percentage of gynoid fat in women with PCOS was comparable to that in healthy controls (SMD -0.07, 95% CI: -0.49-0.35 for percentage). Interestingly, there was no statistically significant difference in all outcomes in MRI or CT subgroup. (**Figure 3** and **Table 2**)

**Table 2:** Meta-analysis results for total body fat, trunk fat, android fat, and gynoid fat in percentage (%): women with PCOS *versus* BMI-matched healthy controls.

Outcomes	No. of cohorts	SMD (95%CI)	*P value*	*I^2^*
Total body fat (%)	13	0.27 (-0.14, 0.69)	0.193	85.6%
Trunk fat (%)	13	0.67 (0.40, 0.94)	<0.001	55.9%
Android fat (%)	11	0.53 (0.12, 0.94)	0.012	84.5%
Gynoid fat (%)	4	-0.07 (-0.49, 0.35)	0.758	65.4%

### Meta-Regression and Subgroup Analyses

Unadjusted meta-regression analyses found that age was inversely associated with visceral fat accumulation in PCOS (P<0.05) (**Table S4**). Subgroup analysis showed that the difference in body fat distribution between PCOS and BMI-matched controls was mainly manifested in non-obese patients. Non-obese PCOS women had elevated accumulation of visceral fat, abdominal subcutaneous fat, total body fat (both in quantity and percentage), trunk fat (in percentage), and android fat (both in quantity and percentage), whereas only trunk fat (in percentage) and android fat (in quantity) were significantly increased in PCOS women with obesity (**Tables S5, S6**). Moreover, subgroup analyses showed that visceral fat and abdominal subcutaneous fat deposition assessed by MRI and CT were similar between women with PCOS and controls regardless of different ethnicities including Caucasian, Asian, and Mediterranean.

### Sensitivity Analyses and Publication Bias

Sensitivity analyses and tests of publication bias verified the robustness of pooled results. No significant variation was introduced in sensitivity analysis for every outcome. Similarly, no significant bias was identified in Egger’s tests and Trim and Filled Analyses (**Table S7**).

## Discussion

This systematic review and meta-analysis initially summarized and compared imaging-based fat distribution between women with PCOS and BMI-matched controls. Higher accumulation of visceral fat, abdominal subcutaneous fat, total body fat, trunk fat, and android fat was observed in women with PCOS, especially non-obese PCOS women. Notably, when imaging method was stratified as the gold standard MRI or CT, there was no difference in fat distribution between women with PCOS and their BMI matched controls.

Based on fat deposition sites and their pathophysiological significance to metabolism, body fat distribution can be generally divided into intra-abdominal/visceral fat (including visceral fat and abdominal subcutaneous fat), upper body fat (including trunk fat and android fat) and lower body fat (gynoid fat). Previous researches have shown that visceral fat and upper body fat are related to higher risks of metabolic disorders such as hypertension and type 2 diabetes, and lower body fat is associated with reduced metabolic risks (61–63). In PCOS, it has been reported that elevated level of testosterone is related to central pattern fat distribution through pro-adipogenic and anti-lipolytic effects, and central obesity in turn aggravates insulin resistance and metabolic complications in women with PCOS (46, 64–66).

Contrary to previous studies which reported an elevated prevalence of central obesity estimated with WC in women with PCOS (9), in this systematic review and meta-analysis, we found that the fat distribution of PCOS patients (including visceral fat, abdominal subcutaneous fat, total body fat, trunk fat, android fat, and gynoid fat) was similar to that of the BMI-matched control group when fat distribution was measured by traditional gold standards MRI or CT. Although these results were inconsistent with the general concept in the field that PCOS patients exhibit visceral fat accumulation, they cannot be simply explained by insufficient sample size since most included studies adopted MRI or CT to evaluate visceral and abdominal subcutaneous fat. Similarly, Mannerås-Holm et al. found in their study that increased abdominal/visceral fat in PCOS women evaluated by waist-to-hip ratio was not supported by MRI, and suggested the need for reassessment of abdominal and visceral fat accumulation in PCOS (12). Moreover, PCOS phenotypes may have impacts on body fat distribution patterns. Aleksandra et al. reported that visceral fat amount was only increased in PCOS phenotype A (hyperandrogenism + oligo/amenorrhea + polycystic ovarian morphology) but not elevated or related to free androgen index in phenotype B (hyperandrogenism + oligo/amenorrhea), C (hyperandrogenism + polycystic ovarian morphology), and D (oligo/amenorrhea + polycystic ovarian morphology), suggesting there are differences in fat distribution between PCOS phenotypes which however is beyond the scope of our study (67). Further studies are therefore needed to clarify the relationship between different PCOS phenotypes and abdominal obesity. Given that PCOS patients with similar BMI or abdominal fat compared to controls also have higher risks of metabolic dysfunctions, our results indicate that central obesity may be independent of PCOS and ectopic fat distribution may not be a dominant reason for high metabolic risks in PCOS (6, 40).

Furthermore, imaging methods may also affect the results. When studies using DXA and ultrasound as measurements were also included in the meta-analysis, the results showed a higher accumulation of total body fat and upper body fat (including visceral fat, abdominal subcutaneous fat, trunk fat, and android fat) in women with PCOS compared to BMI-matched healthy women, which was consistent with previous knowledge. The conflicting results between the different imaging methods may be related to bias from methods. Despite that DXA has been widely used for estimating regional body fat, the results of DXA analysis could be confounded by hydration of lean soft tissue (68). It has been reported that DXA overestimated visceral fat, especially in people who have higher levels of visceral adiposity (69). Therefore, caution should be paid when interpreting the results of DXA in clinical practice.

In meta-regression and subgroup analyses, we found that age was inversely associated with increased visceral fat accumulation in women with PCOS. Similarly, prospective cohort studies have demonstrated that the risks of central obesity and metabolic diseases increased in young PCOS patients but were attenuated in later life (70, 71). However, the underlying mechanism is yet to be elucidated. It is probably due to the protective effect of lifestyle interventions or metformin treatment on PCOS patients. Moreover, subgroup analyses showed that abdominal obesity is more prominent in non-obese patients, suggesting the existence of abnormal body fat distribution in non-obese PCOS phenotype and exercises focused on improving body composition may help prevent and diminish metabolic-related risks in non-obese PCOS women.

This study systematically reviewed the articles that investigated the difference of image-assessed fat distribution between women with PCOS and BMI-matched controls and the robustness of results was verified by sensitivity analyses and tests of publication bias. Through comprehensive subgroup analyses of possible confounders, we found that different imaging methods may be the dominant source of heterogeneity. Given the lack of convenience and efficiency of golden standards MRI and CT, the small sample size is a common problem in studies using MRI or CT to detect fat distribution. This meta-analysis facilitated the integration of these data and found similar abdominal fat distribution between PCOS and BMI-matched healthy controls in MRI or CT subgroup. Despite the above advantages, there are some limitations in this study. Firstly, the main limitation of such an extensive review is that the selection bias in comparator populations. The optimal source of controls should be from the community, but less than half of the included studies met the criteria. In studies that do not use community-based controls, they were more inclined to recruit controls from schools, hospital employees, or medical examiners. This population may be healthier and lead to an overestimation of the difference between PCOS and control groups. Secondly, although details of these possible confounders were extensively extracted from the original studies and displayed in **Table 1** and **Table S2**, subgroup analyses cannot fully explain the heterogeneity. The residual confounding factors may be the definition and phenotype of PCOS, areas measured by imaging methods, lifestyle, and usage of medications. Therefore, further population-based studies with large sample sizes and precise control of confounding factors are still needed.

In conclusion, this systematic review and meta-analysis summarized the current evidence focused on imaging-based body fat distribution in women with PCOS and found similar fat distribution patterns assessed by golden standards MRI or CT between women with PCOS and BMI-matched controls, indicating central obesity may be independent of PCOS and exacerbate metabolic dysregulation in PCOS patients. Moreover, younger patients and non-obese patients were more inclined to accumulate android fat. These results facilitated the understanding of the relationship between PCOS and ectopic fat deposition, and will support the establishment of specialized lifestyle interventions for PCOS patients.

## Data Availability Statement

The original contributions presented in the study are included in the article/**Supplementary Material**. Further inquiries can be directed to the corresponding authors.

## Author Contributions

YL and HY designed the study and evaluated the data. SZ, CH, and ZL collected the information and analyzed the data. SZ, ZL, and FS wrote the manuscript. CW and YL critically revised the manuscript. All authors contributed to the article and approved the submitted version.

## Funding

This study was supported by grants from the Natural Science Foundation of Jiangsu Province (BK20200223), the Natural Science Foundation of Shandong Province (ZR2020QH051), the Young Scholars Program as well as Fundamental Research Funds (21520079614029) of Shandong University to YL.

## Conflict of Interest

The authors declare that the research was conducted in the absence of any commercial or financial relationships that could be construed as a potential conflict of interest.

## Publisher’s Note

All claims expressed in this article are solely those of the authors and do not necessarily represent those of their affiliated organizations, or those of the publisher, the editors and the reviewers. Any product that may be evaluated in this article, or claim that may be made by its manufacturer, is not guaranteed or endorsed by the publisher.
