# Supplementary material for: Imaging-Based Body Fat Distribution in Polycystic Ovary Syndrome: A Systematic Review and Meta-Analysis
Source: Front Endocrinol (Lausanne). 2021 Sep 9;12:697223. doi: 10.3389/fendo.2021.697223 (PMC8458943; doi:10.3389/fendo.2021.697223)
Supplement: Supplementary file 1 [file DataSheet_1.docx]

***Supplemental Table legends***

**Table S1:** Search strategy.

**Table S2:** Additional study characteristics of included studies.

**Table S3:** Assessment of study quality based on Newcastle-Ottawa Scale

**Table S4:** Meta-regression results for visceral fat, abdominal subcutaneous fat, total body fat (both in quantity and percentage), trunk fat (both in quantity and percentage), and android fat (in percentage).

**Table S5:** Subgroup analyses of visceral fat and abdominal subcutaneous fat.

**Table S6:** Subgroup analyses of total body fat, trunk fat, android fat, and gynoid fat.

**Table S7:** Results of sensitive analyses and tests of publication bias.

Table S1: Search strategy.

| **Search strategy of Pubmed** |
| --- |
| ((((magnetic resonance) OR (Imaging, Magnetic Resonance) OR (NMR Imaging) OR (Imaging, NMR) OR (Tomography,NMR) OR (Tomography, MR) OR (MR Tomography) OR (NMR Tomography) OR Zeugmatography OR (Imaging, Chemical Shift) OR (Chemical Shift Imagings) OR (Imagings, Chemical Shift) OR (Shift Imaging, Chemical) OR (Shift Imagings, Chemical) OR (Chemical Shift Imaging) OR (Tomography, Proton Spin) OR (Proton Spin Tomography) OR (Magnetization Transfer Contrast Imaging) OR MRI OR fMRI OR MRIs OR (Spin Echo Imaging) OR (Echo Imaging, Spin) OR (Echo Imagings, Spin) OR (Imaging, Spin Echo) OR (Imagings, Spin Echo) OR (Spin Echo Imagings) OR X-Ray OR (X ray) OR (Computed Tomography) OR Tomodensitometry OR Xray OR (Computerized Tomography) OR Cine-CT OR (Cine CT) OR (Electron Beam Tomography) OR (Beam Tomography, Electron) OR (Tomography, Electron Beam) OR CT OR Ultrasound OR imaging OR Echography OR Ultrasonic OR (Sonography, Medical) OR (Medical Sonography) OR Ultrasounds OR Echotomography OR (Echotomography, Computer) OR (Computer Echotomography))) AND ((((((((((((((((((Body mass distribution[MeSH Terms]) OR body fat distribution[Text Word]) OR Body Fat Patterning[Text Word]) OR adiposity[Text Word]) OR visceral adipose tissue[Text Word]) OR Subcutaneous Adipose Tissue[Text Word]) OR SAT[Text Word]) OR VAT[Text Word]) OR body shape[Text Word]) OR Body composition[Text Word]) OR Adipose tissue[Text Word]) OR fatty tissue distribution[Text Word]) OR adipose tissue distribution[Text Word])) OR Central Obesity[Text Word]) OR Central Obesities[Text Word]) OR Abdominal Obesity[Text Word]) OR Visceral Obesity[Text Word])) AND (((((((((polycystic ovary syndrome[MeSH Terms]) OR Stein Leventhal Syndrome[Title/Abstract]) OR PCOS[Title/Abstract]) OR PCO[Title/Abstract]) OR Polycystic Ovarian Syndrome[Title/Abstract]) OR Polycystic Ovary Syndrome[Title/Abstract]) OR Sclerocystic Ovarian[Title/Abstract]) OR Sclerocystic Ovaries[Title/Abstract]) OR Sclerocystic Ovary[Title/Abstract]) |
| Note: Search strategies of other databases were modified accordingly. |

Table S2: Additional study characteristics of included studies.

| **Author** | **Year** | **Journey** | **Definition of PCOS** | **Adults or Adolescents** | **BMI category** | **Adjusted confounders** | **Medications or treatments usage (YES/NO)** | **Blinded?**  **(YES/NO)** |
| --- | --- | --- | --- | --- | --- | --- | --- | --- |
|  |  |  |  |  |  |  |  |  |
| Barber *et al* | 2008 | JCEM | Rotterdam | Adults | Both | BMI, ethnicity | NO | YES |
| Diaz *et al* | 2018 | Int J Endocrinol | Original definition | Adolescents | Non-obese | Age, BMI | NO | YES |
| Dolfing *et al* | 2011 | Hum Reprod | Rotterdam | Adults | Non-obese | BMI, weight | NO | Not mentioned |
| Jones *et al* | 2012 | JCEM | Rotterdam | Adults | NA | Age, BMI, weight | NO | YES |
| Mannerås-Holm *et al* | 2011 | JCEM | Rotterdam | Adults | Both | Age, BMI, | NO | YES |
| Ezeh *et al* | 2013 | JCEM | NIH | Adults | Both | Age, BMI, ethnicity | NO | Not mentioned |
| Jin *et al* | 2015 | J Obstet Gynaecol Res | Rotterdam | Both | NA | Age, BMI | Not mentioned | Not mentioned |
| Pasquali *et al* | 2000 | JCEM | Original definition | NA | Obese | Age, weight, BMI | NO | Not mentioned |
| Penaforte *et al* | 2010 | J Hum Nutr Diet | Rotterdam | Adults | Obese | Age, ethnicity, weight, BMI | NO | Not mentioned |
| Boumosleh *et al* | 2017 | J Endocr Soc | Rotterdam | Adults | Non-obese; Obese | BMI | NO | Not mentioned |
| Dumesic *et al* | 2016 | JCEM | NIH | Adults | Non-obese | Age, BMI, ethnicity | NO | Not mentioned |
| Echiburú *et al* | 2018 | Steroids | NIH | Adults | Both | Age, BMI | NO | Not mentioned |
| Huang *et al* | 2012 | JCEM | NIH | Adults | NA | Age, BMI | NO | Not mentioned |
| Lourdes *et al* | 2011 | JCEM | NIH | Adolescents | NA | Age, BMI, weight | YES (metformin) | YES |
| Hutchison *et al* | 2011 | JCEM | NIH | Adults | Overweight/Obese | BMI, weight | NO | Not mentioned |
| Kim *et al* | 2018 | Endocr Rev | Original definition | Adolescents | Obese | Age, ethnicity, BMI | NO | Not mentioned |
| Morrison *et al* | 2016 | Hum Reprod | NIH | Adults | Both | Age, BMI, ethnicity | NO | Not mentioned |
| Borruel *et al* | 2013 | JCEM | Rotterdam | Both | Non-obese; Obese | BMI | NO | YES |
| Cascella *et al* | 2007 | Hum Reprod | Rotterdam | Adults | NA | Age, BMI | NO | Not mentioned |
| Jena *et al* | 2018 | Indian J Endocrinol Metab | Rotterdam | Both | Both | Age, BMI | NO | Not mentioned |
| Karabulut *et al* | 2012 | Gynecol Endocrinol | Rotterdam | NA | NA | Age, BMI, ethnicity | NO | YES |
| Moran *et al* | 2014 | JCEM | AE-PCOS | Adults | Non-obese; Obese | Age, BMI | Not mentioned | Not mentioned |
| Sahin *et al* | 2015 | Endocr Pract | Rotterdam | NA | Non-obese; Obese | Age, BMI | NO | YES |
| Tripathy *et al* | 2017 | Eur J Obstet Gynecol Reprod Biol | Rotterdam | Adults | NA | Age, BMI | NO | YES |
| Yildirim *et al* | 2003 | Fertil Steril | NIH | NA | Non-obese | Age, BMI, ethnicity, weight | NO | Not mentioned |
| Braga *et al* | 2018 | Diabetes Metab Syndr | Rotterdam | NA | Both | Age, BMI | NO | Not mentioned |
| Carmina *et al* | 2007 | JCEM | NIH | NA | Obese; Overweight; Normal weight | Age, BMI, weight | NO | Not mentioned |
| Cree-Green *et al* | 2017 | J Endocr Soc | NIH | Adolescents | Non-obese | Age, BMI | NO | Not mentioned |
| Cree-Green *et al* | 2016 | Obesity | NIH | Adolescents | Obese | Age, BMI | NO | Not mentioned |
| Cunha *et al* | 2019 | Clin Nutr. | Rotterdam | Adults | Both | Age, BMI | NO | Not mentioned |
| Faloia *et al* | 2004 | J Endocrinol Invest | NIH | NA | Non-obese; Obese | Age, BMI | NO | Not mentioned |
| Glintborg *et al* | 2016 | Acta Obstet Gynecol Scand | Rotterdam | Adults | Both | Age, BMI, ethnicity | NO | Not mentioned |
| Godoy-Matos *et al* | 2009 | Gynecol Endocrinol | Rotterdam | Adults | NA | Age, BMI | NO | Not mentioned |
| González *et al* | 2014 | JCEM | NIH | NA | Non-obese; Obese | Age, BMI | NO | Not mentioned |
| Good *et al* | 1999 | Fertil Steril | NIH | NA | Non-obese | Age, BMI, ethnicity, weight | NO | Not mentioned |
| Jȩdrzejuk *et al* | 2016 | Gynecol Endocrinol | Rotterdam | Adults | NA | Age, BMI, weight | NO | Not mentioned |
| Kirchengast *et al* | 2004 | Fertil Steril | Original definition | Adults | Non-obese | Age, BMI, ethnicity | NO | Not mentioned |
| Kogure *et al* | 2014 | Gynecol Endocrinol | Rotterdam | Adults | Obese; Overweight; Normal weight | Age, BMI | NO | Not mentioned |
| Macruz *et al* | 2017 | Int J Gynaecol Obstet | Rotterdam | Both | Non-obese | Age, BMI, weight | NO | Not mentioned |
| Mierzwicka *et al* | 2018 | Gynecol Endocrinol | Rotterdam | Adults | Both | BMI | NO | Not mentioned |
| Pepene *et al* | 2012 | Clin Endocrinol | AE-PCOS | NA | Overweight/Obese | Age, BMI | NO | Not mentioned |
| Satyaraddi *et al* | 2019 | J Hum Reprod Sci | Rotterdam | Adults | Non-obese; Obese | Age, BMI | NO | Not mentioned |
| Schmidt *et al* | 2012 | Clin Endocrinol | Rotterdam | Adults | Both | Age, BMI, weight | Not mentioned | Not mentioned |
| Shroff *et al* | 2007 | JCEM | Rotterdam | Adults | Obese | Age, BMI | NO | YES |
| Thomann *et al* | 2008 | Gynecol Endocrinol | Rotterdam & NIH | NA | NA | Age, BMI | NO | Not mentioned |
| Toscani *et al* | 2007 | Metab: Clin Exp | Original definition | Both | Overweight/Obese | Age, BMI, hirsutism | NO | Not mentioned |
| Yucel *et al* | 2006 | Eur J Obstet Gynecol Reprod Biol | Rotterdam | NA | Both | Age, BMI | NO | YES |

BMI: Body mass index; NA: Not available;

| **Author** | **Year** | **Total** | **Selection** | | | | **Comparability** | **Outcome** | | |
| --- | --- | --- | --- | --- | --- | --- | --- | --- | --- | --- |
|  |  |  | **S1** | **S2** | **S3** | **S4** | **C1C2** | **O1** | **O2** | **O3** |
| Cunha *et al* | 2019 | 7 | * |  | * | * | ** |  | * | * |
| Satyaraddi *et al* | 2019 | 7 | * |  | * | * | ** |  | * | * |
| Braga *et al* | 2018 | 6 | * |  |  | * | ** |  | * | * |
| Diaz *et al* | 2018 | 7 |  | * |  | * | ** | * | * | * |
| Echiburú *et al* | 2018 | 6 | * |  |  | * | ** |  | * | * |
| Jena *et al* | 2018 | 7 | * | * |  | * | ** |  | * | * |
| Kim *et al* | 2018 | 6 | * |  |  | * | ** |  | * | * |
| Mierzwicka *et al* | 2018 | 6 | * | * |  | * | * |  | * | * |
| Boumosleh *et al* | 2017 | 5 |  |  | * | * | * |  | * | * |
| Cree-Green *et al* | 2017 | 6 | * |  |  | * | ** |  | * | * |
| Macruz *et al* | 2017 | 7 | * | * |  | * | ** |  | * | * |
| Tripathy *et al* | 2017 | 8 | * | * |  | * | ** | * | * | * |
| Cree-Green *et al* | 2016 | 6 | * |  |  | * | ** |  | * | * |
| Dumesic *et al* | 2016 | 9 | * | * | * | * | ** | * | * | * |
| Glintborg *et al* | 2016 | 8 | * | * | * | * | ** |  | * | * |
| Jȩdrzejuk *et al* | 2016 | 7 | * | * |  | * | ** |  | * | * |
| Morrison *et al* | 2016 | 7 | * | * |  | * | ** |  | * | * |
| Jin *et al* | 2015 | 6 | * | * |  | * | * |  | * | * |
| Sahin *et al* | 2015 | 6 | * |  |  | * | * | * | * | * |
| González *et al* | 2014 | 7 | * | * |  | * | ** |  | * | * |
| Kogure *et al* | 2014 | 7 | * | * |  | * | ** |  | * | * |
| Moran *et al* | 2014 | 8 | * | * | * | * | * | * | * | * |
| Borruel *et al* | 2013 | 7 | * | * |  | * | * | * | * | * |
| Ezeh *et al* | 2013 | 8 | * |  | * | * | ** | * | * | * |
| Huang *et al* | 2012 | 7 | * |  | * | * | ** |  | * | * |
| Jones *et al* | 2012 | 8 | * | * |  | * | ** | * | * | * |
| Karabulut *et al* | 2012 | 9 | * | * | * | * | ** | * | * | * |
| Pepene *et al* | 2012 | 6 | * | * |  | * | * |  | * | * |
| Schmidt *et al* | 2012 | 6 | * |  | * |  | ** |  | * | * |
| Dolfing *et al* | 2011 | 6 | * | * |  | * | * |  | * | * |
| Hutchison *et al* | 2011 | 7 | * | * | * | * | * | * | * |  |
| Lourdes *et al* | 2011 | 9 | * | * | * | * | ** | * | * | * |
| Mannerås-Holm *et al* | 2011 | 9 | * | * | * | * | ** | * | * | * |
| Penaforte *et al* | 2010 | 7 | * | * |  | * | ** |  | * | * |
| Godoy-Matos *et al* | 2009 | 7 | * | * |  | * | ** |  | * | * |
| Barber *et al* | 2008 | 8 | * | * | * | * | * | * | * | * |
| Thomann *et al* | 2008 | 6 | * | * |  | * | * |  | * | * |
| Carmina *et al* | 2007 | 7 | * | * |  | * | ** |  | * | * |
| Cascella *et al* | 2007 | 6 | * | * |  | * | * |  | * | * |
| Shroff *et al* | 2007 | 8 | * | * |  | * | ** | * | * | * |
| Toscani *et al* | 2007 | 7 | * | * |  | * | ** |  | * | * |
| Yucel *et al* | 2006 | 8 | * | * |  | * | ** | * | * | * |
| Kirchengast *et al* | 2004 | 6 | * | * |  | * | * |  | * | * |
| Faloia *et al* | 2004 | 6 | * |  |  | * | ** |  | * | * |
| Yildirim *et al* | 2003 | 7 | * | * |  | * | ** |  | * | * |
| Pasquali *et al* | 2000 | 8 | * | * | * | * | * | * | * | * |
| Good *et al* | 1999 | 7 | * | * |  | * | ** |  | * | * |

Table S3: Assessment of study quality based on Newcastle-Ottawa Scale

Table S4: Meta-regression results for visceral fat, abdominal subcutaneous fat, total body fat (both in quantity and percentage), trunk fat (both in quantity and percentage), and android fat (in percentage).

| **Outcomes** | **Age** | | **BMI** | |
| --- | --- | --- | --- | --- |
|  | **Coefficients (95%CI)** | ***P value*** | **Coefficients (95%CI)** | ***P value*** |
| Visceral fat | -0.05 (-0.08, -0.02) | 0.005 | -0.03 (-0.07, 0.006) | 0.094 |
| Abdominal subcutaneous fat | -0.02 (-0.05, 0.006) | 0.109 | -0.003 (-0.03, 0.02) | 0.815 |
| Total body fat | 0.004 (-0.04, 0.04) | 0.827 | -0.02 (-0.06, 0.01) | 0.242 |
| Total body fat (%) | 0.05 (-0.08,0.17) | 0.426 | -0.04 (-0.14,0.06) | 0.389 |
| Trunk fat | -0.10 (-0.26, 0.06) | 0.189 | -0.02 (-0.10, 0.05) | 0.490 |
| Trunk fat (%) | 0.002 (-0.3, 0.13) | 0.979 | -0.01 (-0.08, 0.07) | 0.814 |
| Android fat (%) | 0.07 (-0.08,0.21) | 0.305 | -0.07 (-0.20,0.06) | 0.282 |

Meta-regression analyses were only performed on outcomes with above 10 included studies.

Outcomes marked with (%) refer to outcomes expressed in percentage, and outcome without (%) mark refer to outcomes expressed in quantity.

BMI: Body mass index;

Table S5: Subgroup analyses of visceral fat and abdominal subcutaneous fat.

| **Subgroups** | **Visceral fat** | | | **Abdominal subcutaneous fat** | | |
| --- | --- | --- | --- | --- | --- | --- |
|  | **SMD (95%CI)** | ***P value*** | ***I^2^*** | **SMD (95%CI)** | ***P value*** | ***I^2^*** |
| **Obese** |  |  |  |  |  |  |
| Overall | 0.28 (-0.21, 0.76) | 0.264 | 72.1% | 0.25 (-0.06, 0.54) | 0.109 | 0.0% |
| MRI or CT | 0.09 (-0.38, 0.57) | 0.701 | 52.7% | 0.15 (-0.32,0.62) | 0.529 | 0.0% |
| **Non-obese** |  |  |  |  |  |  |
| Overall | 0.71 (0.28,1.14) | 0.001 | 68.7% | 0.54 (0.33,0.76) | <0.001 | 0.0% |
| MRI or CT | 0.55 (-0.43, 1.52) | 0.272 | 76.9% | 0.59 (0.17,1.00) | 0.005 | 0.0% |
| **Adults** |  |  |  |  |  |  |
| Overall | 0.38 (0.16,0.60) | 0.001 | 65.9% | 0.26 (0.11,0.40) | 0.001 | 19.4% |
| MRI or CT | 0.13 (-0.10, 0.36) | 0.278 | 17.6% | 0.16 (-0.06,0.38) | 0.158 | 0.0% |
| **Caucasian** |  |  |  |  |  |  |
| Overall | 0.23 (0.04,0.42) | 0.019 | 37.9% | 0.28 (0.11,0.45) | 0.001 | 12.7% |
| MRI or CT | 0.15 (-0.09,0.39) | 0.219 | 36.2% | 0.15 (-0.06,0.36) | 0.161 | 0.0% |
| **Asian** |  |  |  |  |  |  |
| Overall | 0.65 (0.19,1.11) | 0.006 | 88.3% | 0.24 (0.07,0.40) | 0.005 | 39.4% |
| MRI or CT | -0.05 (-0.34,0.24) | 0.730 | -- | 0.06 (-0.23,0.35) | 0.690 | -- |
| **Mediterranean** |  |  |  |  |  |  |
| Overall | 0.74 (0.43,1.05) | <0.001 | 24.7% | 0.48 (0.26,0.70) | <0.001 | 45.6% |
| MRI or CT | 0.55 (-0.28,1.37) | 0.193 | 69.1% | 0.37 (-0.06,0.81) | 0.091 | 81.7% |

Table S6: Subgroup analyses of total body fat, trunk fat, android fat, and gynoid fat.

| **Subgroups** | **Total body fat** | | | **Total body fat (%)** | | |
| --- | --- | --- | --- | --- | --- | --- |
|  | **SMD (95%CI)** | ***P value*** | ***I^2^*** | **SMD (95%CI)** | ***P value*** | ***I^2^*** |
| Obese | 0.12 (-0.09,0.32) | 0.256 | 0% | 0.13 (-0.99,1.25) | 0.820 | 92.0% |
| Non-obese | 0.38 (0.15,0.61) | 0.001 | 68.8% | 0.67 (0.07,1.27) | 0.028 | 72.3% |
| Adults | 0.32 (0.13,0.52) | 0.001 | 68.5% | 0.55 (-0.14,1.24) | 0.115 | 92.2% |
| Caucasian | 0.12 (-0.04,0.28) | 0.144 | 5.7% | 0.02 (-0.26,0.31) | 0.880 | 60.6% |
| Asian | 0.47 (0.16,0.79) | 0.003 | 90.5% | 1.48 (1.14,1.83) | 0.000 | 0.0% |
| Mediterranean | 0.17 (-0.18,0.52) | 0.339 | 60.9% | - | - | - |
| **Subgroups** | **Trunk fat** | | | **Trunk fat (%)** | | |
|  | **SMD (95%CI)** | ***P value*** | ***I^2^*** | **SMD (95%CI)** | ***P value*** | ***I^2^*** |
| Obese | 0.40 (-0.09,0.90) | 0.112 | 54.2% | 0.62 (0.14,1.09) | 0.011 | 37.5% |
| Non-obese | 0.51 (-0.01,1.03) | 0.054 | 66.4% | 0.84 (0.31,1.37) | 0.002 | 69.8% |
| Adults | 0.67 (-1.15,2.48) | 0.472 | 92.6% | 0.33 (-0.05,0.71) | 0.089 | 26.3% |
| Caucasian | 0.46 (0.12,0.80) | 0.007 | 57.3% | 0.70 (0.41,0.99) | 0.000 | 59.2% |
| Asian | - | - | - | - | - | - |
| Mediterranean | 0.58 (0.02,1.14) | 0.042 | - | - | - | - |
| **Subgroups** | **Android fat** | | | **Android fat (%)** | | |
|  | **SMD (95%CI)** | ***P value*** | ***I^2^*** | **SMD (95%CI)** | ***P value*** | ***I^2^*** |
| Obese | 0.35 (0.02,0.67) | 0.040 | 0.0% | -0.20 (-1.61,1.12) | 0.775 | 88.6% |
| Non-obese | 0.90 (0.58,1.21) | 0.000 | 0.0% | 1.22 (0.91,1.52) | 0.000 | 0.0% |
| Adults | 0.24 (-0.08,0.56) | 0.147 | 0.0% | 0.34 (-0.36,1.04) | 0.336 | 87.9% |
| Caucasian | 0.51 (0.22,0.81) | 0.001 | 46.7% | 0.53 (0.09,0.98) | 0.018 | 86.1% |
| Asian | -0.03 (-0.32,0.25) | 0.814 | -- | -- | -- | -- |
| Mediterranean | -0.28 (-1.03,0.47) | 0.459 | -- | 0.50 (-0.26,1.26) | 0.197 | -- |
| **Subgroups** | **Gynoid fat** | | | **Gynoid fat (%)** | | |
|  | **SMD (95%CI)** | ***P value*** | ***I^2^*** | **SMD (95%CI)** | ***P value*** | ***I^2^*** |
| Obese | -- | -- | -- | -- | -- | -- |
| Non-obese | 0.14 (-0.82,1.10) | 0.770 | -- | -0.80 (-1.79,0.19) | 0.115 | -- |
| Adults | -0.10 (-0.45,0.25) | 0.581 | 0.0% | -0.22 (-0.66,0.22) | 0.327 | 58.8% |
| Caucasian | -0.10 (-0.45,0.25) | 0.581 | 0.0% | -0.07 (-0.49,0.35) | 0.758 | 65.4% |
| Asian | 0.43 (0.01,0.86) | 0.021 | -- | -- | -- | -- |
| Mediterranean | 0.22 (0.03,0.42) | 0.043 | -- | -- | -- | -- |

Total body fat, trunk fat, android fat and gynoid fat could be expressed in quantity or in percentage. Outcomes marked with (%) refer to outcomes expressed in percentage, and outcome without (%) mark refer to outcomes expressed in quantity.

Table S7: Results of sensitive analyses and tests of publication bias.

| **Outcome** | **No. of cohorts** | **Effects Model** | **Variations in Sensitivity Analyses** | **Egger's Test for Publication Bias**  **(*P value*)** | **Variations in Trim and Filled Analyses**  **SMD (95%CI)** |
| --- | --- | --- | --- | --- | --- |
| Visceral fat | 24 | Random | NS | 0.821 | - |
| Abdominal subcutaneous fat | 23 | Fixed | NS | 0.896 | - |
| Total body fat | 20 | Fixed | NS | 0.794 | - |
| Total body fat (%) | 13 | Random | NS | 0.946 | - |
| Trunk fat | 10 | Random | NS | 0.520 | - |
| Trunk fat (%) | 13 | Random | NS | 0.029 | NS |
| Android fat | 9 | Random | NS | 0.415 | - |
| Android fat (%) | 11 | Random | NS | 0.435 | - |
| Gynoid fat | 5 | Fixed | NS | 0.334 | - |
| Gynoid fat (%) | 4 | Random | NS | 0.691 | - |

-: no trimming performed; NS: No significant variations introduced;

Outcomes marked with (%) refer to outcomes expressed in percentage, and outcome without (%) mark refer to outcomes expressed in quantity.
